# Progress in Surgery

**Published:** 1900-05-05

**Authors:** 


### PROGRESS IN SURGERY.

### DISEASES OF THE BLADDER AND PROSTATE GLAND.

**Cystitis.**—N. Senn,[1] discussing the ætiology and classification of cystitis, remarks that it is now generally conceded that inflammation of any tissue or organ is invariably caused by microbic invasion, and that other causes act only by favouring infection, and therefore an accurate knowledge of the nature of the microbic infection is essential in the successful treatment of cystitis. The predisposing causes either effect tissue changes, and so determine the localisation of microbes, or they furnish a nutrient medium for the growth of the organisms. Among the predisposing causes are retention of urine, unrest of the bladder, abnormal urine, tumours, calculus, foreign bodies, pressure from without or within, exposure to cold, venous stasis, and trauma. Microbic infection may occur by the urethra, the urine, adjacent organs, or the blood. Four methods of classification are applicable: 1. Anatomical: (*a*) pericystitis, (*b*) paracystitis, (*c*) interstitial cystitis, (*d*) endocystitis. 2. Pathological: embracing the catarrhal, suppurative, ulcerative, exudative, and exfoliative forms of cystitis. 3. Clinical: Acute and chronic cystitis. 4. Bacteriological: The organism may be the bacillus coli, saphrophytic, staphylococcus, streptococcus, streptococcus erysipelatis, typhoid bacillus, diplobacillus, gonococcus, or tubercle bacillus. Senn recommends that in all long-standing cases of cystitis a careful bacteriological examination of the urine should be made. Ramon Guitéras[2] states that cases of irritable bladder are often called cystitis, though really they are nothing more than the result of congestion of the neck of the bladder, from a foreign body or from the irritating character of the urine. In the same article the cause of acute cystitis is said to be the extension of gonorrhœa from the urethra during the third or fourth week, or to instrumentation in a case of posterior urethritis. Acute cystitis may be confounded with posterior urethritis, prostatitis, seminal vesiculitis, and vesical irritability. In posterior urethritis the pus would flow back into the bladder. When the urine was passed into three glasses the last glass would contain more pus in cystitis than in posterior urethritis. In the latter affection the first glass would contain more pus than the other two, and also shreds. The localisation of the seat of tenderness and swelling by rectal examination would distinguish prostatitis and vesiculitis. In acute cystitis hot sitz baths twice daily, as hot as can be borne, are recommended. Also hot rectal saline douches. By the mouth, alkaline diluents and antispasmodics should be given, and suppositories of belladonna and morphine. For bladder irrigation the solution of permanganate of potash is advised of the strength of 1:4,000 at first, later up to 1:1,000. Subsequently nitrate of silver solution should be used, using first 1:16,000, and slowly increasing to 1:4,000. Sometimes it is better to use deep urethral instillations of nitrate of silver, using solutions of a strength varying from one to four grains to the ounce. When the urine was alkaline nothing in the way of intestinal medication was equal to urotropine, given in doses of ʒss. three or four times a day. W. K. Otis[2] recommended when there was posterior urethritis, as well as cystitis, that the catheter should be introduced only just within the external sphincter. The fluid syringed in then would not escape from the catheter, and the patient should be allowed to void the fluid, thereby cleansing both urethra and bladder at the same time. The double-way catheter, he stated, did not efficiently serve to cleanse the bladder, the fluid passing quickly from one to the other eye of the instrument. Total emptying of the bladder was most successfully accomplished if the patient stood up for the purpose; the erect posture should therefore be assumed when washing out if the patient were strong enough.

**Lithotomy.**—The editors of the *Lancet*[3] quote important statistics bearing upon the choice of operation for the cure of vesical calculus. Their remarks preface the notes of a case of suprapubic lithotomy contributed by Vincent Jackson. It is pointed out that lateral lithotomy has been so infrequently performed in recent years that no extensive statistics are available. Those collected by C. Williams, and founded on the cases at the Norfolk and Norwich Hospital from 1772 to 1869, appear to be the best. In that period 871 operations were performed, and of these 116 resulted in death, yielding a death rate of 13·32 per cent. In boys the results were better still, for between the ages of five and ten years there was a mortality of less than 4 per cent. Probably more modern statistics would show some improvement on these results, but would not probably show the mortality to be less than half that in the above statistics. The mortality of the modern operation of litholopaxy may be gauged from the following statistics: R. Harrison performed 101 litholopaxies from 1890 to 1897 and had six deaths, *i.e.*, a little less than 6 per cent. R. Baker, I.M.S., has published notes of 404 cases, with a mortality under ½ per cent. Thus the mortality of litholopaxy varies, but is certainly less than that in lateral lithotomy. Recurrence is more frequent after the crushing operation, but with a much lower mortality this is not of great importance. The results in the high operation vary. Freyer has stated that operation in children has a mortality of 20 per cent. Witte had a mortality of 12·5 per cent. in 32 cases, and Lissjanski that of 5·5 per cent. in 18 cases. Lithotomy is generally considered to be the best operation for stones of large size; but Milton has said: "The larger the stone, and the more exhausted the patient, the greater is the indication for lithotrity." Every method gives more favourable results in boys than in men. Vincent Jackson's case was quite an ordinary one, and very successful. The bladder and the abdominal wound were completely closed at once. A plea for lateral lithotomy in boys in suitable cases is raised.

**Ectopia Vesicæ.**—D. E. Mundell[4] briefly reviews the methods by which it has been sought to relieve this distressing condition, and makes the suggestion that a portion of a sheep's bladder be transplanted for the purpose of making an anterior wall for the incomplete bladder. There have been two lines of operative treatment pursued; one, plastic, by means of skin-flaps; the other, placing the urinary receptacle deeper in the pelvis, either by diverting the urinary passages or by approximating the innominate bones. Trendelenburg approximates the bones, König performs osteotomy of the pubic bones. In both the urine is in contact with the line of junction of the symphysis, and interferes with union. In the operation for diverting the urinary channels towards the rectum there is great risk of infection of the kidney by ascending ureteritis. Also, usually, the urine acts as an irritant to the rectal mucous membrane. Gersuny converted the rectal pouch into a receptacle for urine only, by making an artificial anus and shutting off the upper end of the rectum entirely. Rutkowski, instead of using the skin as a flap, brought down a portion of the intestine which he had first excised, but left still receiving its vascular supply through the attached mesentery, and sutured it to the edges of the defective bladder. The *rationale* of this is to remedy the defect in a muscular organ by a flap containing muscle. Skin flaps used for the purpose have many defects. The hairs on the inturned skin favour calcareous deposits; the macerating action of the urine sets up inflammation; and the absence of any sphincter-like muscle necessitates the constant use of a pad. Mundell found that he could transplant a part of a dog's bladder into the subcutaneous tissue of another dog interposing a sheet of gold foil between its mucous surface, turned downwards, and the subjacent subcutaneous tissue. The results were satisfactory. He now, therefore, suggests a mode of dealing with ectopia vesicæ, viz., to transplant a portion of a sheep's bladder to the lower lateral abdominal fascia of the patient. Let it remain there seven or eight days until union occurs. Then perform a plastic operation, whereby a skin flap with the bladder attached may be swung over upon the extruded bladder, and its edges sutured thereto. The fate of the transplanted bladder tissue will be to lose its normal structure if left too long buried in the subcutaneous tissues.

[1] Med. Chron., Dec., 1899. [2] Med. Rec., Jan., 1900. [3] Lancet, Jan., 1900. [4] Annals of Surg., Dec., 1899.

